# Exploring Gas Evolution Oscillators: Mechanisms and Applications

**DOI:** 10.1002/cphc.202400841

**Published:** 2024-11-29

**Authors:** Marcello A. Budroni, Mauro Rustici, Federico Rossi

**Affiliations:** ^1^ Department of Chemical, Physical, Mathematical and Natural Sciences University of Sassari Via Vienna 2 07100 Sassari Italy; ^2^ Department of Physical Sciences Earth and Environment University of Siena Piazzetta Enzo Tiezzi 1 53100 Siena Italy

**Keywords:** chemical oscillators, gas evolution dynamics, phase transition instabilities, nonlinear chemical kinetics, supersaturation, nucleation phenomena

## Abstract

We review an iconic class of chemical oscillators driven by phase transition instabilities, namely Gas Evolution Oscillators (GEOs). These systems show oscillatory dynamics in the delivery of gas sustained simple reactions yielding gaseous products in a liquid mixture, due to nucleation and supersaturation phenomena. After presenting the main features and properties of these systems, we deepen the underlying mechanism through a unified picture of the various models that have been proposed to describe this kind of oscillations. We finally discuss a concrete example of how such instabilities can impact chemical processes with applied relevance.

## Graphical abstract

Explore the rhythms of gas evolution oscillators, i. e. systems showing oscillatory delivery of gas sustained by simple reactions yielding gaseous products in a liquid mixture, due to nucleation and supersaturation phenomena. We delve into their mechanisms and their potential to control chemical processes, with a focus on sustainable energy applications.

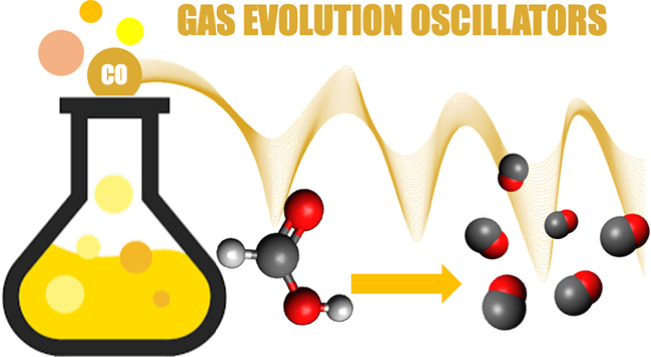

## Introduction

Understanding oscillatory phenomena in chemical systems is of paramount importance for approaching fundamental aspects of biological complexity and to control and optimize many processes with applied relevance.[^1^, ^2^, ^3^]

In homogeneous phase, chemical systems typically undergo oscillatory instabilities in far‐from‐equilibrium conditions (either under a constant flux of the reactants or in batch when the reactants consume slowly with respect to the lifespan of intermediates) due to nonlinear kinetics involving fast activatory steps combined to relaxation steps through which the system can restore pre‐autocatalysis conditions (so‐called “resetting of the chemical clock”) on a slower time scale. These scenarios have been widely investigated and reviewed.[^3^, ^4^, ^5^, ^6^]

Chemical oscillations can also develop in the presence of simple nonoscillatory reactions that can trigger and couple with physically‐induced oscillatory instabilities, resulting in periodic changes of the spatio‐temporal chemical composition. In these systems, chemical oscillations are therefore the result of shifting from kinetic to physical nonlinearities.^[7]^ Representative classes in this context are chemically‐driven hydrodynamic instabilities and phase transitions. Autonomous emergent oscillatory dynamics were recently obtained with A+B→ C reactions thanks to a nonlinear synergy between this non‐oscillatory kinetics and convection.[^8^, ^9^, ^10^, ^11^] In heterogeneous systems, spatial and temporal oscillations characterize precipitation reactions in the form of periodic bands of precipitate as it happens in the Liesegang phenomenon,[^12^, ^13^, ^14^] in the budding growth of chemical gardens,^[15]^ in traveling precipitation fronts[^16^, ^17^] and in simple dissolution processes in the presence of aggregated systems.^[18]^

Here we focus on the case of *nucleation‐phase oscillators* and, in particular, to gas evolution oscillators (GEO), where one or more gaseous species are rapidly generated by a chemical process within a homogeneous solution and the resulting gas is delivered with periodic bursts rather than at a smooth rate.[^4^, ^19^, ^20^, ^21^] This behavior, first observed in 1916 during the acid‐catalyzed decomposition of formic acid into H_2_O and CO,^[22]^ regained interest in the 1980s through detailed studies framed within the emerging field of nonlinear chemistry. Despite this, a systematic and comprehensive review of these phenomena has yet to be undertaken.

In the following sections, we first detail the main properties and experimental characteristics of the phenomenon (Sec. Phenomenology). Then, through a numerical approach, we unify the various models that have been proposed over time to describe GEOs, offering a cohesive understanding of their mechanisms (Sec. Modeling). This part includes comprehensive information and practical guidance for replicating typical experimental and simulated behaviors associated with these systems. We finally discuss the possible impact of this kind of instability on a practical application in the context of green energy, namely the hydrolysis of borohydride salts, which is a promising process for the generation of gaseous molecular hydrogen to be used as a sustainable vector of energy (Sec. Applied Aspects).

## Phenomenology

The first and most famous example of GEOs is the Morgan reaction,^[22]^ which exhibits pronounced bursts in the CO flux during the acid‐catalyzed decomposition of formic acid into H_2_O and CO, according to the scheme:
(1)

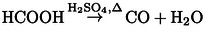




**Figure 1:**
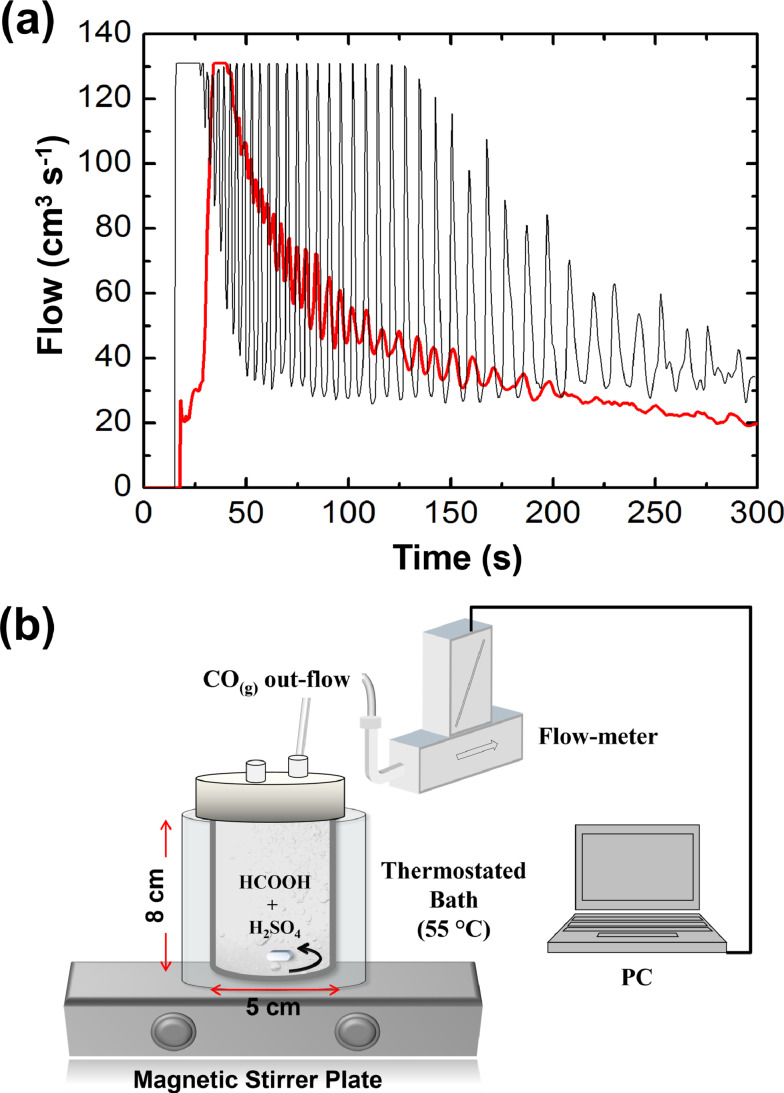
(a) Typical profiles of gaseous CO outflow during the Morgan reaction carried out with 2 ml of HCOOH 98 % and 4 ml H_2_SO_4_ 98 % at 55 °C,, under stirring at 500 rpm. The red and the black traces compare the system behavior with and without glass powder in the reaction environment. Clearly, the presence of glass introduces nucleation points and reduces supersaturation effects at the basis of the phenomenon, thus decreasing the oscillations amplitude. Figure adapted with permission from Ref. [23]. (b) Sketch of the experimental setup used to perform the reaction.

Figure 1(a) shows a typical trace of the CO outflow during this process carried out by pouring 2 ml of HCOOH 98 % into 4 ml H_2_SO_4_ 98 % at 55 °C and stirring at 500 rpm. The reaction is performed in batch conditions, within a 150 ml flask immersed in a thermostated bath and stirring the reactive solution with a small magnetic bar moved by a programmable stirrer, as sketched in the setup of Figure 1(b). The evolving gas is piped through a flowmeter to record the related dynamics. The temporal profile of the CO outflow shows regular bursts with a period ranging from the 4–5 s of the initial regime to the 12–13 s characterizing the final oscillations. During each burst, the solution turns milky from the formation of numerous small bubbles, causing it to foam up quickly; successively, the foam relaxes and the solution follows a quiescent period with a very low evolution of the gas. In general, the frequency and the pattern of these oscillations are very sensitive to changes in the experimental conditions. The oscillations exhibited by the Morgan reaction are sustainable over extended periods and occur across a broad spectrum of reactant concentrations and temperatures. For reference, periodic bursts have been observed within a temperature range of 20 °C to 65 °C, with formic acid concentrations between 1 and 4 M, and sulfuric acid concentration exceeding 70 %. Importantly, the onset of this oscillatory instability is critically dependent upon stirring: in unstirred or very slightly stirred solutions, bursts of gas evolution are quite erratic while vigorously stirred solutions generate gas smoothly without any rhythms.

In a series of works in the late 1970s and 80s, Noyes and co‐workers reconsidered the analysis of this system and extended an analogous exploration to several other chemical reactions able to produce gaseous species in homogeneous solution. Indeed, oscillatory behaviors in the gas outflow were found to be a rather recurrent phenomenon in the decomposition of malic, oxalic, tartaric, citric, and malonic acids in concentrated sulfuric acid generating oxides of carbon,^[24]^ decomposition of benzene diazonium salts,^[25]^ decomposition of hydrogen peroxide,^[26]^ as well as during the development of nitrogen from the reaction of aqueous nitrous acid with both ammonium ion and urea.[^27^, ^28^] However, due to its relatively higher reproducibility compared to other GEOs, the Morgan reaction has become the primary focus of investigation for understanding the mechanisms behind these oscillatory phenomena.

The common features of different GEOs were characterized by measuring the key parameters involved in the control of the oscillatory instability^[29]^ and combined to understand whether oscillations stemmed from a positive feedback of the foaming on the reaction kinetics^[19]^ or were due to a chemical “oscillophor” within the homogeneous phase driving a pulsated release of one or more gaseous products of the reaction.^[20]^ Examples of the latter case are the repetitive glows in the production of electronically excited CO_2_ during the oxidation of carbon monoxide or cool flames during the reaction of hydrocarbons with oxygen, both due to the thermal feedback on the chemical kinetics. These thermokinetic oscillations, mostly occurring in gaseous reactions, were extensively studied by Gray et al. and a detailed analysis can be found in Ref. [3]. The famous Belousov‐Zhabotinsky reaction itself gives rise to small oscillations in the evolution of CO_2_ (which is one of the ultimate products of the reaction) coordinated to the redox oscillations in the solution. Following this line, Showalter and Noyes proposed a tentative description of the GEOs based on a complicated nonlinear kinetic model.[^4^, ^20^]

However, a thorough experimental analysis led to attribute unambiguously the oscillations in GEOs to a physical mechanism based on the nucleation of bubbles in supersaturated solutions.[^4^, ^27^, ^30^, ^31^] More specifically, as it takes energy to create a surface between the liquid and the gas phase, supersaturation of the dissolved gas may become extremely large before a fluctuation can produce bubbles; when this happens, bubbles grow and collectively escape the solution by rising in the gravitational field with the characteristic foaming. There is then a period of quiescence until the critical supersaturation for a new burst is attained again. GEOs were thus classified as “Phase‐Nucleation Oscillators”.^[4]^

## Modeling

### The M‐Variable Bubblelator

On the basis of the interpretative picture described above, Smith and Noyes[^30^, ^31^] developed a model relying on the “life‐cycle” of a population of bubbles with different sizes, which nucleation and growth is fed *in‐situ* by an almost constant input of molecules of the dissolved gas provided by the reactive source. The model involves five main steps: (1) the reaction (decomposition of formic acid for the Morgan system) generates a smooth input of molecules of dissolved gas (carbon monoxide) into the homogeneous solution; (2) the solution becomes progressively more supersaturated but remains metastable until it attains a critical concentration at which spontaneous nucleation begins almost discontinuously; (3) the initial growth of small bubbles has little impact on the solution, and there is a period during which dissolved gas molecules are still being formed faster than how they are being removed by the growing bubbles and a large reservoir of bubble nuclei is formed; (4) as major bubble growth begins, the solution is depleted of dissolved gas more rapidly than it can be produced by chemical reaction. Nucleation is no longer possible, and the smaller existing bubbles redissolve; (5) when the bubble growth has almost stopped, the solution is cleared because existing bubbles rise and escape. This mechanism repeats itself cyclically.

To simplify the system formalization, the authors made a series of assumptions. First, Henry's law valid and the ideal gas law applicable in the bubbles, where the pressure is considered uniform. Since the bubble surface relaxes rapidly, the equilibrium surface tension was used and applied independently of the bubble size. Moreover, the rate of bubble growth was accounted by transport across the bubble surface and not by diffusion in the bulk solution, since moderate stirring and upward rising of the bubbles prevent concentration gradients in the solution around a bubble. Coalescence of bubbles, which would provide another growth pathway for small bubbles, was considered secondary than individual growth, and thus ignored. Finally, bubbles with different sizes were assumed to behave identically and escape the solution with equal probability.

The resulting model, the so‐called “Bubblelator”,[^30^, ^31^] consists in the following set of coupled differential equations in the main variables describing the state of the system, namely the concentration of the dissolved gas, *C_bulk_*, the difference between the released gas and atmospheric pressures, $\Delta P=P_{gas}-P_{atm}$ , and the concentrations of bubbles with different radius partitioned in *M* classes, i. e. *N_j_* (with $j\;\in \;\left[1,M\right]$ ):

(2) $$\frac{dC_{bulk}}{dt}=\Phi_{react}-\frac{A}{V_{s}}k_{ex}\left(C_{bulk}-C_{sat}\right)\;\;\;-\frac{4\pi r_{1}^{3}}{3RT}P_{1}J_{n}-\sum_{j=1}^{M}4\pi r_{j}^{2}k_{tr}N_{j}\left(C_{bulk}-\kappa P_{j}\right)$$

(3) $$\frac{d\Delta P}{dt}=v_{esc}F-k_{cap}\Delta P$$

(4) $$\mathrm{with}\;v_{esc}=k_{ex}A\left(C_{bulk}-C_{sat}\right)+\sum_{j=1}^{M}\frac{4\pi r_{j}^{3}P_{j}k_{j}V_{s}N_{j}}{3RT}$$

(5) $$\mathrm{if}\;r_{j}>r_{eg}\;\;\frac{dN_{j}}{dt}=J_{n}-\left(q_{j}+k_{j}\right)N_{j}\;\mathrm{for}\;j=1\;\;\frac{dN_{j}}{dt}=q_{j-1}N_{j-1}-\left(q_{j}+k_{j}\right)N_{j}\;\mathrm{for}\;1<j<M\;\;\frac{dN_{j}}{dt}=q_{j-1}N_{j-1}-k_{j}N_{j}\;\mathrm{for}\;j=M$$

(6) $$\mathrm{if}\;r_{j}>r_{eg}\;\;\frac{dN_{j}}{dt}=q_{j+1}N_{j+1}-\left(q_{j}+k_{j}\right)N_{j}\;\mathrm{for}\;j\neq M\;\;\frac{dN_{j}}{dt}=-\left(q_{j}+k_{j}\right)N_{j}\;\mathrm{for}\;j=M$$

(7) $$\mathrm{if}\;r_{j}=r_{eg}\;\;\frac{dN_{j}}{dt}=-k_{j}N_{j}\forall_{j}$$

In Eq. (2), the first negative term describes evaporation of molecules from the surface of the stirred solution. The second negative term refers to the loss of molecules by formation of nuclei, while the summation, which individual contributions may be positive or negative, accounts for the interchange of molecules between bulk solution and bubbles of various sizes. Φ_*react*_ is the rate of the chemical reaction producing molecules of dissolved gas (here assumed as a system constant), which can be evaluated by measuring either the pressure change due to the evolved gas or the change in composition of the residual solution; typical values for the Morgan reactions were measured in Ref. [32]. *A* and *V_s_* are the area of surface and the volume of the reactive solution. *k_ex_* is the rate constant for transport of molecules from the surface of the stirred solution to the gas phase. *C_sat_* is the gas concentration in equilibrium with pressure of the gas above the solution, *P_gas_*, according to $C_{sat}=\kappa P_{gas}$ , being *κ* the Henry's law constant. $r_{1}=\frac{2\kappa \sigma }{C_{crit}-C_{sat}^{0}}$ is the radius of bubbles in equilibrium with the critical concentration of gas for bubbles nucleation at atmospheric pressure, *C_crit_* (*σ* is the surface tension of the bubbles in equilibrium with the solution and $C_{sat}^{0}=\kappa P_{atm}$ ). *k_tr_* is the rate constant for transport of molecules between bubble and solution. $J_{n}$ =$\alpha \;\exp[-\beta /\left(C_{bulk}-C_{crit}{)}^{2}\right]$ describes the rate of formation of nuclei of radius *r*_1_ ^[30]^ (*α* and *β* are adjustable parameters from the classic nucleation theory).^[33]^ However, for practical use, *J_n_* can be approximated to a step function, i. e. *J_n_* equals a constant if $C_{bulk}>C_{crit}$ and is null otherwise. *R* and *T* are the gas universal constant and the temperature, respectively. *P*_1_ is the pressure of bubbles characterized by radius *r*_1_ and, more in general, *P_j_* is the pressure of a bubble with radius, *r_j_*, falling in the *j*‐th interval of the *M*‐partitioned set (see further explanation below), as expressed by the relation.[^27^, ^30^, ^33^]

(8) $$P_{j}=P_{gas}-2\sigma /r_{j}$$

Eq. (3) governs the change of pressure ΔP in the reactor due to (*i*) the transfer of gas from the solution and (*ii*) the possible gas escape through a capillary leak (if any), controlled by rates *v_esc_* and *k_cap_*, respectively. The term *v_esc_*, defined by Eq. (4), is thus the total rate of transfer of gaseous molecules from the solution to the gas phase either by evaporation or by escape of bubbles of various sizes; $F=760\frac{\mathrm{torr}}{\mathrm{atm}}\;82.053\;\frac{{\mathrm{cm}}^{3}\mathrm{atm}}{\mathrm{atm}\;K}\frac{T}{V_{s}}\frac{K}{{\mathrm{cm}}^{3}}$ is a conversion factor used to relate moles of gas to the pressure generated in the flask. Eqs. (5–6) describe the rate of change in the concentration of those bubbles with radius larger (Eq. (5) or smaller Eq. (6)) than the reference radius *r_eq_* of a bubble in equilibrium with *C_bulk_* ($r_{eq}=\frac{2\kappa \sigma }{C_{bulk}-C_{sat}}$ ). The former tend to grow while the latter shrink. Eq. (7) expresses the physical escape of those bubbles with no net tendency either to grow or to shrink. In this context key parameters are the growth (or shrink) rate constants, *q_j_*, for the *j*‐th class of bubbles given by:

(9) $$q_{j}={\frac{1}{\Delta }}_{j}\left|\frac{dr}{dt}\right|=\frac{1}{\Delta_{j}}\left|\frac{3RTk_{tr}r_{j}\left(C_{bulk}-C_{sat}^{0}-2\kappa \sigma /r_{j}\right)}{3P_{\mathrm{atm}}\;r_{j}+4\sigma }\right|\;$$

Since bubbles radii can range from 10^−6^ cm for small nuclei to 0.1 cm characterizing large bubbles, the *j*‐th radius increment in the radii discretization, Δ_*j*_, was conveniently taken to be constant on a logarithmic scales, i. e.:

(10) $$\Delta_{j}=r_{1}[\left(r_{M}/r_{1}{)}^{1/2\left(M-1\right)}-1\right]\;\;\mathrm{for}\;j=1$$

(11) $$\Delta_{j}=r_{1}{\left(r_{M}/r_{1}\right)}^{\left(j-3/2\right)/\left(M-1\right)}[\left(r_{M}/r_{1}{)}^{1/\left(M-1\right)}-1\right]\;\mathrm{for}\;j\;\in \left(1,M\right)$$

(12) $$\Delta_{j}=r_{M}-r_{1}{\left(r_{M}/r_{1}\right)}^{\left(M-3/2\right)/\left(M-1\right)}\;\mathrm{for}\;j=M$$

where $r_{j}=r_{1}{\left(r_{M}/r_{1}\right)}^{\left(j-1\right)/\left(M-1\right)}\;\;\;\forall \;\;1<j<M$ . *k_j_* are the rate constants for the escape of bubbles of radius *r_j_*, quantified by the expression $k_{j}=g\rho r_{j}^{2}/3\mu \bar{z}$ , where *g*, *μ* and $\bar{z}$ are the gravitational acceleration, the dynamic viscosity and the average position of the bubble below the surface, respectively.

**Figure 2:**
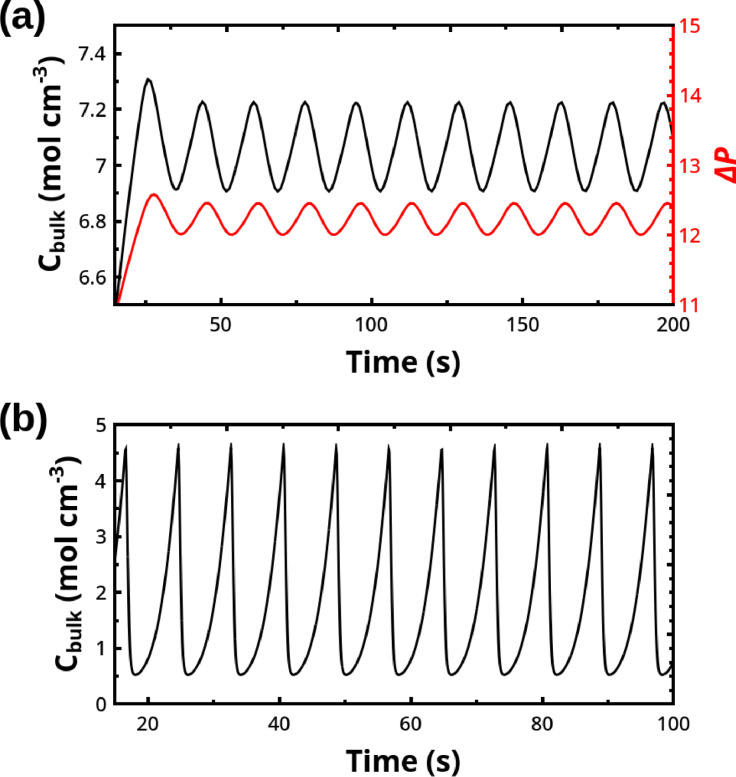
(a) Numerical solution of Eqs. (2–7) by means of the Euler method with the integration time step $h_{t}=10^{-4}$ s. Parameter setting: M=60 ; $\Phi_{react}=3.1\times 10^{-6}$ cm^−3^ s^−1^; $\kappa =1.08\times 10^{-9}$ mol cm^−3^ torr^−1^; σ=0.041 torr cm; A=29.8 cm^2^; $V_{g}=178$ cm^3^; $V_{s}=28$ cm^3^; $k_{ex}=0.031$ cm s^−1^; $C_{crit}=7\times 10^{-5}$ mol cm^−3^; $C_{sat}^{0}=8.2\times 10^{-7}$ mol cm^−3^; $r_{1}=2\kappa \sigma /\left(C_{crit}-C_{sat}\right)$ cm; $r_{M}=0.1$ cm; $R=6.236\times 10^{4}$ torr cm^3^ mol^−1^ K^−1^; T=313.15 K; $P_{atm}=760$ torr; $k_{s}=0.05$ s^−1^; $k_{tr}=k_{ex}/V_{s}=0.1$ cm s^−1^; $J_{n}=10000$ cm^−3^ s^−1^ if $C_{bulk}>C_{crit}$ mol cm^−3^ and 0 otherwise; g=980 cm s^−2^; $k_{cap}=0.579$ s^−1^; μ=0.145 g cm^−1^ s^−1^; ρ=1.8 g cm^−3^; $\bar{z}=0.595$ cm; initial conditions are $C_{bulk}=4.5\times 10^{-5}$ mol cm^−3^, ΔP=0 torr, $N_{j}=0$ ∀j cm^−3^. (b) Numerical solution of Eqs. (13–15) by means of the 4‐*th* order explicit Runge‐Kutta method with integration time step $h_{t}=10^{-4}$ and the following parameter setting: $\Phi_{reac}=3.1\times 10^{-5}$ mol cm^−3^; $r_{0}=1.74\times 10^{-6}$ cm; $r_{b}=10^{-1}$ cm; $\kappa =10^{-12}$ mol dyn^−1^ cm^−1^; $C_{crit}=7\times 10^{-5}$ mol cm^−3^; $C_{sat}=10^{-6}$ mol cm^−3^, $R=8.314\times 10^{7}$ erg mol^−1^ K^−1^; T=313.15 K; $k_{g}=0.8$ s^−1^; $k_{s}=0.05$ s^−1^; σ=60 dyn cm^−1^; $k_{e}=0.7$ s^−1^; $k_{tr}=0.1$ cm s^−1^; $J_{n}=10000$ cm^−3^ if $C_{bulk}>C_{crit}=7\times 10^{-5}$ and 0 otherwise; initial conditions are $C_{bulk}=4.5\times 10^{-5}$ mol cm^−3^, X=Y=0 cm^−3^.

Figure 2(a) shows numerical simulations of Eqs. (2–7) carried out by means of a classic Euler method and using the experimental values detailed in the figure's caption. A general guide for the implementation of a program for the numerical simulation of this system are reported in Ref. [34].

### The 3‐Variable Bubblelator

Although the Bubblelator provides a satisfactory semi‐quantitative picture of the oscillatory phenomena in GEOs,^[32]^ it presents a large number of parameters and equations (*M*>20 is needed to generate stable solutions) which hinders an analytical approach. In this perspective, Noyes proposed a reduced scheme of the Bubblelator in three global variables, namely the concentration of the dissolved gaseous molecules, *C_bulk_*, the concentration of the nuclei, *X*, and that of the bubbles, *Y*. The resulting model reads:^[35]^

(13) $$\frac{dC_{bulk}}{dt}=\Phi_{react}-k_{s}\left(C_{bulk}-C_{sat}\right)\;\;\;-\frac{4\pi r_{0}^{3}}{3\kappa RT}\left(C_{sat}+2\kappa \sigma /r_{0}\right)J_{n}\;\;\;-4\pi r_{b}^{2}k_{tr}\left(C_{bulk}-C_{sat}-2\kappa \sigma /r_{b}\right)Y$$

(14) $$\frac{dX}{dt}=J_{n}-k_{g}X$$

(15) $$\frac{dY}{dt}=k_{g}X-k_{e}Y$$

Eq. (13) for *C_bulk_* is essentially analogous to that of the (*M*+2)‐variable model above, except that pressure and concentration terms have been rewritten using Eq. (8) and Henry's law. The three negative terms in Eq. (13) describe the transfer of dissolved molecules from the surface of the solution to the gas phase (referring to the previous formulation $k_{s}=\frac{A}{V_{s}}k_{ex}$ ), the conversion of molecules to nuclei of radius *r*_0_, and the release of dissolved molecules in bubbles of radius *r_b_*, respectively. The first‐order rate constants, *k_g_* and *k_e_*, refer to the growth of a nucleus to a bubble and the escape of a bubble from the solution, respectively. A numerical solution of this system is shown in Figure 2(b), where an oscillatory regime is obtained by simulating Eqs. (13–15) with the 4^*th*^‐order Runge–Kutta method and the parameters detailed in the caption, mostly of which corresponding to those used for simulating model (2–7).

### Delayed Equation Model

The “life‐cycle” of bubbles in this phenomenon also suggested an alternative and mathematically elegant approach to reduce the description into a single equation.^[36]^ Nuclear bubbles, formed at a rate *J_n_* as determined by the instantaneous concentration of dissolved molecules, take some time before rising rapidly to the surface and discharging the dissolved molecules into the surrounding atmosphere. This time lag, *t*_0_, somehow accounting for the effect of the *M* equations that govern the growth of nuclei and bubbles in Eqs. (5–7) (and, in turn, the quiescent period before the bursting escape of the gas) can be included into the equation for *C_bulk_* to regulate dynamically the nucleation rate term as follows:

(16) $$\frac{dC_{bulk}\left(t\right)}{dt}=\Phi_{react}-k_{\nu }\left(C_{bulk}\left(t\right)-C_{sat}\right)\;\;\;\;\times \;\exp[-\beta /\left(C_{bulk}\left(t-t_{0}\right)-C_{sat}{)}^{2}\right]$$

where the rate constant $k_{\nu }$ cannot be rigorously defined in terms of experimentally measurable quantities. A direct comparison with the experimental observable, i. e. the system pressure *P*, is obtained through the equation:

(17) $$\frac{dP\left(t\right)}{dt}=k_{2}\;\left(C_{bulk}\left(t\right)-C_{sat}\right)\;\exp[-\beta /\left(C_{bulk}\left(t-t_{0}\right)-C_{sat}{)}^{2}\right]\;\;\;-k_{fl}\left(P\left(t\right)-P_{0}\right)$$

in which the first term accounts for the pressure increment due to escape of gas from the solution, while the second term describes the leakage of gas, if any, from the system to the external atmosphere of constant pressure *P*_0_, as controlled by the first‐order rate constant *k_fl_* (this is null for sealed reactors).

By using the scaled quantities, $x=\left(C_{bulk}\left(t\right)-C_{sat}\right)/\beta^{\frac{1}{2}}$ , $\tau =\Phi_{react}\;t/\beta^{\frac{1}{2}}$ , $k=k_{\nu }\;\beta^{\frac{1}{2}}/\Phi_{react}$ , $p=\left(P\left(t\right)-P_{0}\right)\;k_{\nu }/\left(k_{2}\beta^{\frac{1}{2}}\right)$ , $k_{1}=k_{fl}\beta^{\frac{1}{2}}/\Phi_{react}$ , Eqs. (16–17) reduce to the simpler dimensionless form:

(18) $$\frac{dx\left(\tau \right)}{d\tau }=1-k\;x\left(\tau \right)\;\exp[-1/x\left(\tau -\tau_{0}{)}^{2}\right]$$

(19) $$\frac{dp\left(\tau \right)}{d\tau }=k\;x\left(\tau \right)\;\exp[-1/x\left(\tau -\tau_{0}{)}^{2}\right]-k_{1}\;p\left(\tau \right)$$

The resolution of this delayed differential equation (DDE) system requires the definition of an initial history function for $x\left(\tau \right)$ (i. e. the values assumed by the delayed variable in the temporal range $\tau \in \left[-\tau_{0},0\right)$ ), which is here fixed to zero (this is physically consistent with the system at hands, but other choices scarcely affect the asymptotic dynamics of this problem). DDEs (18–19) can be directly integrated by means of an explicit 4^*th*^‐order Runge‐Kutta method, by storing the history function dynamically in a buffer array with dimension INT($\tau_{0}/h_{t}$ ) (being *h_t_* the integration time step), from which the values of the delayed variable at time $\tau -\tau_{0}$ can be called and updated during the integration.

**Figure 3:**
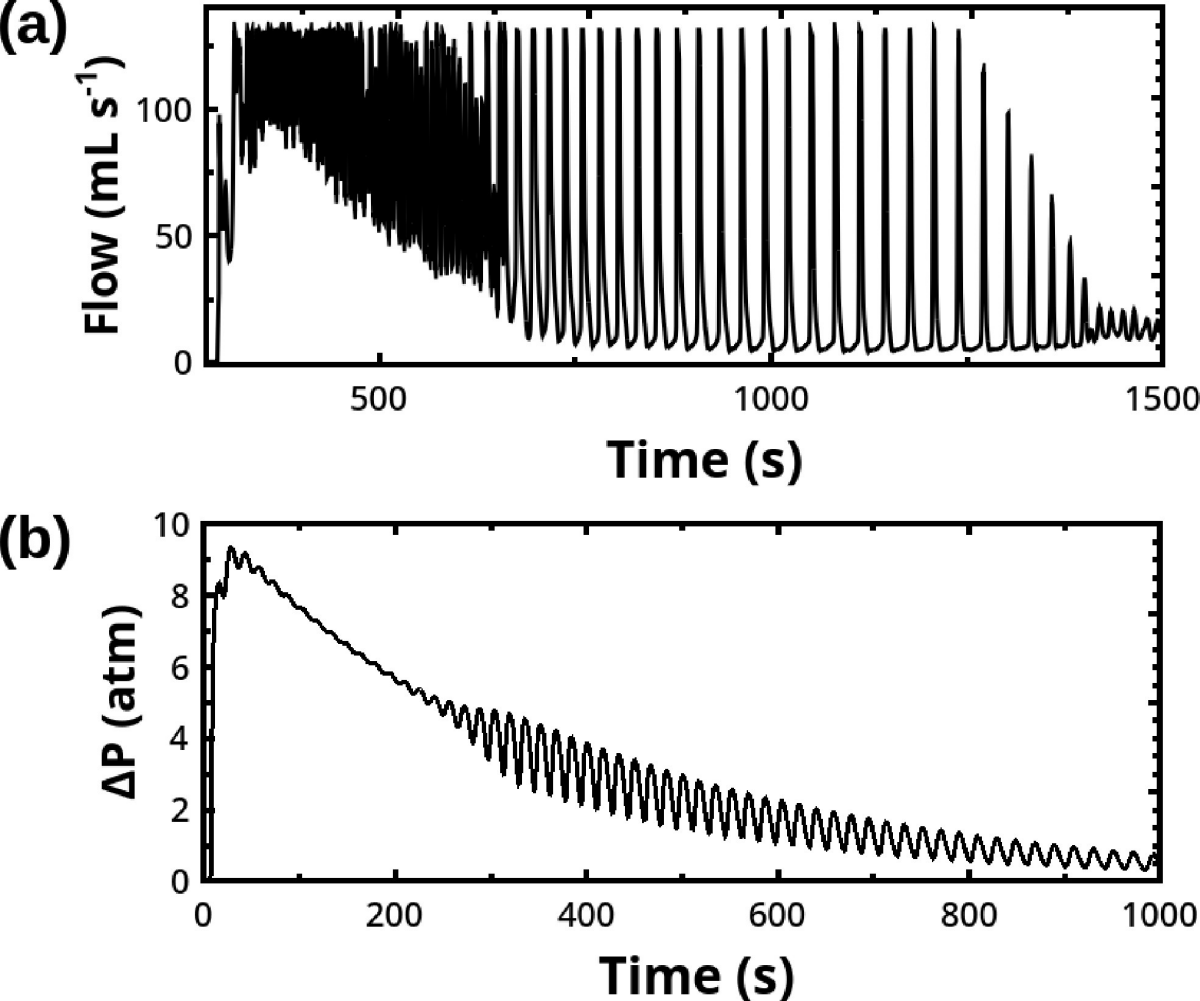
(a) Oscillatory pattern traced by the Morgan reaction carried out with 2 ml of HCOOH 98 % into 4 ml H_2_SO_4_ 98 % at 55 °C and stirring at 200 rpm. (b) Simulation of a GEO *via* numerical integration of Eqs. (18–19) with $\tau_{0}=6$ s, k=1 , *k*_1_ *=*0.1, and fixed integration time step $h_{t}=10^{-4}$ . $x\left(\tau \right)$ and $p\left(\tau \right)$ are initially set to zero.

Eqs. (18–19) formalize a strongly approximated view of the phenomenon: the single delay *τ*_0_ has to be chosen *ad‐hoc* to best fit experiments and cannot be simply and specifically correlated with any experimental quantity since there are diverse possible sources of delay. Nevertheless, this formulation finds a good qualitative agreement with a wide range of experimental behaviors shown by GEOs. As an illustrative example, in Figure 3 we compare a typical oscillatory pattern exhibited by the Morgan reaction (see conditions in the caption) and a numerical simulation of Eqs. (18–19) with $\tau_{0}$ =6 s.

## Applied Aspects

Apart from their obvious fundamental interest in the realm of nonlinear dynamics and oscillatory instabilities, the oscillatory release of a gas can influence processes of practical interest. An example is the production of molecular hydrogen to be used as an alternative green energy vector from compounds where it is chemically stored and released on‐demand. In this context, the hydrolysis of borohydride salts represents a promising process for the generation *in‐situ* of pure molecular hydrogen. Hydrogen can be converted into electrical power in combination with PEM fuel cells thanks to its oxidation with air in mild conditions, giving water as the main by‐product. Among borohydride systems, sodium borohydride, NaBH_4_, is conveniently adopted because of its considerable hydrogen gravimetric capacity, high stability in air, and relatively low price. Hydrogen generation *via* NaBH_4_ hydrolysis, globally described by the reaction:^[37]^

(20) $${\mathrm{NaBH}}_{4}\left(\mathrm{aq}\right)+4H_{2}O\to \mathrm{NaB}{\left(\mathrm{OH}\right)}_{4}\left(\mathrm{aq}\right)+4H_{2}\left(g\right)+\mathrm{heat},$$

spontaneously occurs in mild conditions^[38]^ and can be promoted by an acidic environment. The reaction yields environmentally safe by‐products (in particular borates that can be re‐cycled).

In their former studies, Noyes and coworkers tested, among the others, this system to check possible oscillatory dynamics in the outflow of the gas like that exhibited by GEOs. Though they could not find any evidence for oscillatory regimes, they did not exclude the possibility of such behaviors in this system.^[4]^ Indeed, the reaction can present high complexity in the development of the gas including long‐lasting periodic and more complicated oscillations as shown in several studies where the process is integrated in pilot models for energy generation.[^39^, ^40^, ^41^]

**Figure 4:**
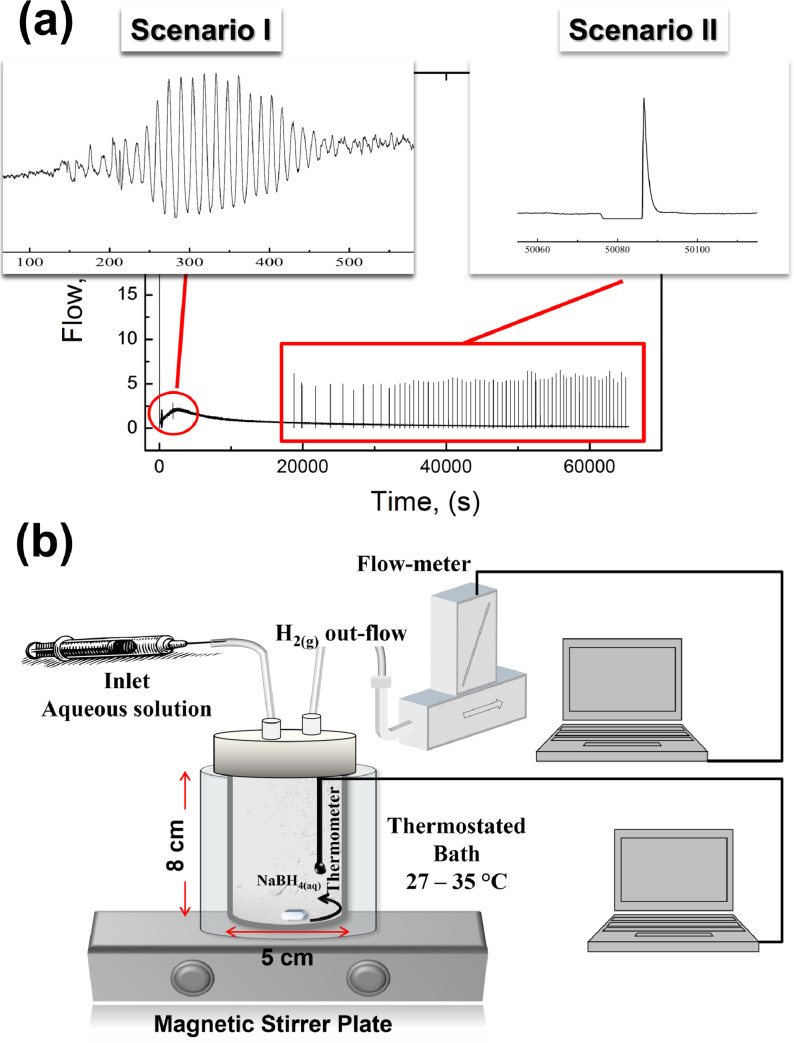
(a) Example of gaseous H_2_ out‐flow in the non‐catalyzed NaBH_4_ hydrolysis carried out with 1.5 g of NaBH_4_ and 15 ml of distilled water (i. e. NaBH_4_=2.6 M) at 30 °C and stirring at 250 rpm. The dynamics can show two typical oscillatory scenarios: a sinusoidal‐type transient and a bursting‐type regime. Flow is given in standard cubic centimeters per minute (sccm). (b) Setup used to perform the experiment. Figure adapted with permission from Ref. [43]. Copyright 2017 American Chemical Society.

Examples of these behaviors are reported in Figure 4 with two typical oscillatory scenarios of gas delivery that can develop during the NaBH_4_ hydrolysis. One is a sinusoidal‐type periodic transient that takes place at the very beginning of the process (after 150–200 s). This regime typically consists of around 10 oscillations with a characteristic period of 18±2 s and a small amplitude ranging between 0.5 and 2 cm^3^ s^−1^. The second scenario is a long‐lasting bursting‐type regime in which each oscillation pattern shows an initial decrease of the gas‐flow, followed by a spike and, then, a relaxation phase. The amplitude of these oscillations is much larger than that observed in the sinusoidal‐type scenario, ranging between 5 and 20 cm^3^ s^−1^ in mild laboratory conditions.

Although the integral amount of the evolving gas may be not severely affected by this phenomenon, the fluctuating dynamics of gas delivery may impact the performance of a system fueled by the borohydride hydrolysis, making the transformation of hydrogen into electrical power unstable. Periodic behaviors may also evolve into more complex and undesirable dynamical regimes, such as intermittent bursting and chaotic oscillations.[^42^, ^43^] Thus, mastering conditions for the onset of the oscillatory instability may allow a chemical control and stabilization of the gas outflow and, in turn, of the power generation.

Despite the clear impact that such oscillatory instabilities may present in practical terms, they have been somehow overlooked from the fundamental viewpoint and a robust picture to explain these phenomena is still missing.

Recent studies were devoted to identify the main control parameters governing the onset and the properties of the oscillatory outflow. Though these phenomena suffered of scarce reproducibility, the salt concentration, the working temperature, the stirring rate, the solution pH and the material of the reactor were isolated as key factors.[^23^, ^43^] More specifically, oscillations occur beyond a threshold of the salt concentration around 1 M. The increment of the salt concentration goes along with an increase in the oscillation frequency and amplitude. Analogously, high temperatures increase the oscillation frequency and the flow baseline. The dependence on the temperature is critical for the bursting‐like scenario which was only observed for temperature values larger than 30 °C. An optimal range of the stirring rate values is necessary in order to observe regular flow oscillations: the gas flow traces a noisy monotonic pattern for stirring rates lower than 250 rpm while pushing agitation beyond 1000 rpm determines a slight reduction of the oscillations amplitude. Periodic behaviors could be reproduced successfully in the pH range [5.00–8.00] and the use of the NaH_2_PO_4_/Na_2_HPO_4_ buffer allowed to stabilize the bursting‐like scenario. Although oscillations maintain a non‐stationary character over a decreasing baseline, the length of the bursting transient increases by increasing the initial pH (provided it is below 9) and becomes maximal around pH=7. This effect weakens when decreasing the buffering strength.

Many key dependence of this oscillator, particularly the critical sensitivity upon the stirring, the temperature and reactant concentration, appear clearly consistent with GEOs. Other characteristics, like the exothermic nature of the reaction combined to the dependence of the phenomenon on the reactor material, could point to a nonlinear thermal feedback on the kinetics combined with the heat dissipation. By means of “ad‐hoc experiments”, recent works[^23^, ^43^] aimed at disentangling whether oscillatory gas release were powered by these physical mechanisms. These studies could exclude the possible involvement of a thermokinetic interplay and suggested that they are most probably driven by a master chemical “oscillophor” in the reactive mixture. More specifically, the classical kinetic scheme used to describe the reaction:[^44^, ^45^, ^46^]
(21)

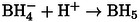



(22)

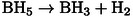



(23)

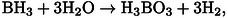




was implemented by hypothesizing a cubic autocatalysis:
(24)

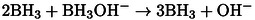




based on the experimental evidence that the intermediate BH_3_OH^−^ forms during the reaction.^[43]^ This step was assumed as the global result of the equilibrium between BH_3_ and B_2_H_6_ coupled with an isotopic exchange between the latter and BH_3_OH^−^,
(25)

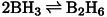



(26)

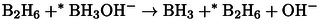




and supposing that step (25) develops significantly faster than step (26), which allows to apply the standard adiabatic approximation,^[47]^ d[B_2_H_6_]/d*t*=0.

Though this could represent a minimal model to explain oscillations in the system, a definitive response about the role of supersaturation could not be given, and all the considerations presented in this review should be taken into account for a chemo‐physical control of this process.

## Concluding Remarks

Physically‐driven chemical oscillations significantly impact numerous chemical processes in both fundamental and applied sciences. In this paper we have reviewed one class of systems where chemical oscillations are sustained by a transition phase started and sustained by a reaction able to produce gaseous products. Gas evolution oscillators essentially rely on a nonlinear interplay between gas nucleation and supersaturation phenomena. After a general picture on the history and main experimental features reported in the literature (here reproduced *ad‐hoc*), we have described the mechanism triggering the periodic release of gas from the reactive mixture through a systematic and comparative analysis of the models proposed for the phenomenon.

Finally, we have reported on a concrete case where the oscillatory instability characterizing GEOs can challenge the regular delivery of gas during the hydrolysis of borohydride salts, which is considered a viable process for the delivery on‐demand of chemically‐stored molecular hydrogen to be used in combination with fuel cells for power generation.

As we have seen for borohydride hydrolysis, it could be difficult to disentangle the origin of complexity in chemical processes involving phase transition instabilities, as they may combine with other sources of nonlinearities, like chemical feedbacks. Another exemplifying case is the Bray‐Liebhafsky (BL) reaction,[^48^, ^49^] a prototypical chemical oscillator showing homogeneous batch oscillations during the iodate–catalyzed decomposition of hydrogen peroxide in acidic solution. In this system, oscillations in the concentration of iodine, iodide and oxygen result in a periodic switch of the reaction mixture color from pale yellow to blue‐purple. For long time this phenomenon has been described in terms of an autocatalytic chemical mechanism in homogeneous phase, involving a number of intermediates like I, HOI, HIO_2_ and HOO⋅, and summarized by two overall steps the alternately dominate the dynamics:[^50^, ^51^]

(27) $$2{\mathrm{IO}}_{3}^{-}+5H_{2}O_{2}+2H^{+}\to I_{2}+5O_{2}\left(g\right)+6H_{2}O$$

(28) $$I_{2}+5H_{2}O_{2}\to {\mathrm{IO}}_{3}^{-}+2H^{+}+4H_{2}O$$

Despite the large number of works attempting at discerning the intimate mechanism of the BL reaction, many aspects of the system, such as the sensitivity to the stirring, pressure, oxygen concentration, remain unexplained and under current debate. In this context, Stanisavljev et al. have recently proposed a variant to existing kinetic models, by including the possible influence of heterogeneous effects like the nucleation of bubbles, in line with the theoretical framework reviewed in this paper.^[52]^ In particular, they suggested that the local energy of fast collapse of unstable nuclei of oxygen bubbles, forming in saturated conditions, can couple with the chemical kinetics, providing the driving force for critical and thermodynamically unfavorable substeps in the iodine oxidation reaction (28).

Similarities between the gas evolution oscillators and the calcium phosphate oscillator can be also pointed out. In the latter, a periodic precipitation of calcium phosphate occurs under stirring, induced by a nonlinear enzymatic reaction, the hydrolysis of urea by enzyme urease, which produces an autocatalytic increase of the pH and, in parallel, of phosphate ions, successively consumed by the burst nucleation of the precipitation when the phosphate concentration exceeds supersaturation.^[53]^

In general, this transport‐driven and other kinds of physically‐driven instabilities can play a role in numerous fundamental and practical problems and, thus, their control is of crucial interest for chemists and chemical engineers communities.^[7]^

## Conflict of Interests

There are no conflicts of interest to declare.
